# Unconventional Constituents and Shared Molecular Architecture of the Melanized Cell Wall of *C. neoformans* and Spore Wall of *S. cerevisiae*

**DOI:** 10.3390/jof6040329

**Published:** 2020-12-01

**Authors:** Christine Chrissian, Coney Pei-Chen Lin, Emma Camacho, Arturo Casadevall, Aaron M. Neiman, Ruth E. Stark

**Affiliations:** 1 CUNY Institute for Macromolecular Assemblies, City University of New York, New York, NY 10031, USA; cchrissian@ccny.cuny.edu; 2 Department of Chemistry and Biochemistry, The City College of New York, New York, NY 10031, USA; 3 Ph.D. Program in Biochemistry, The Graduate Center of the City University of New York, New York, NY 10016, USA; 4 Department of Biochemistry and Cell Biology, Stony Brook University, Stony Brook, NY 11794, USA; coneylin@gmail.com; 5 Department of Molecular Microbiology and Immunology, Johns Hopkins Bloomberg School of Public Health, Johns Hopkins University, Baltimore, MD 21205, USA; ecamach2@jhmi.edu (E.C.); acasade1@jhu.edu (A.C.); 6 Ph.D. Program in Chemistry, The Graduate Center of the City University of New York, New York, NY 10016, USA

**Keywords:** fungal cell wall, solid-state NMR, macromolecular assembly, *Saccharomyces cerevisiae*, *Cryptococcus neoformans*, dityrosine, melanin, triglycerides, chitin and chitosan

## Abstract

The fungal cell wall serves as the interface between the cell and the environment. Fungal cell walls are composed largely of polysaccharides, primarily glucans and chitin, though in many fungi stress-resistant cell types elaborate additional cell wall structures. Here, we use solid-state nuclear magnetic resonance spectroscopy to compare the architecture of cell wall fractions isolated from *Saccharomyces cerevisiae* spores and *Cryptococcus neoformans* melanized cells. The specialized cell walls of these two divergent fungi are highly similar in composition. Both use chitosan, the deacetylated derivative of chitin, as a scaffold on which a polyaromatic polymer, dityrosine and melanin, respectively, is assembled. Additionally, we demonstrate that a previously identified but uncharacterized component of the *S. cerevisiae* spore wall is composed of triglycerides, which are also present in the *C. neoformans* melanized cell wall. Moreover, we identify a tyrosine-derived constituent in the *C. neoformans* wall that, although it is not dityrosine, is a non-pigment constituent of the cell wall. The similar composition of the walls of these two phylogenetically distant species suggests that triglycerides, polyaromatics, and chitosan are basic building blocks used to assemble highly stress-resistant cell walls and the use of these constituents may be broadly conserved in other fungal species.

## 1. Introduction

The fungal cell wall is an architecturally complex and dynamic structure that serves as the interface between the cell and its surroundings and has myriad roles that are essential for cell viability, integrity, and in pathogenic species, for virulence [1,2]. The structural foundation of the cell wall is an interwoven network of polysaccharides that serves as a scaffolding for the deposition of smaller quantities of auxiliary components such as pigments, proteins and lipids, together forming a protective barrier [3]. The bulk of the polysaccharides are glucans and chitin, which are found in varying proportions and molecular arrangements in different species, so that each species elaborates its own unique cell wall architecture [4]. Despite its complexity and robustness, the fungal cell wall is far from inert; it undergoes constant change, both during normal processes such as cell budding and growth and also in response to environmental stressors [5]. The cell wall is able to respond to changes in conditions such as temperature, pH, osmotic pressure, and nutrient availability by rapidly remodeling to better withstand these potentially harmful conditions. This ability to react to external stimuli is a critical component of the stress adaptation response that enables fungal organisms to colonize a wide array of disparate niches, including infecting animal hosts [6]. Indeed, the dynamic adaptability of the cell wall is a key contributor to virulence in pathogenic fungal species; as the initial point of contact between host and microbe, the rapid restructuring of the cell wall is a primary strategy fungal pathogens use to protect against host defense mechanisms and evade the immune response during infection [7,8]. As fungal infections are a worldwide problem associated with high morbidity and mortality rates [9], the cell wall has been an active topic in research for several decades, including as a target for antifungal therapeutic discovery.

*Cryptococcus neoformans* is a basidiomycete fungus and the causative agent of life threatening cryptococcal meningoencephalitis in immunocompromised individuals [10]. When presented with certain exogenous aromatic substrates in a nutrient limited environment, the *C. neoformans* cell wall that is primarily composed of glucans and chitin is augmented by the deposition of melanin pigments, a response essential for virulence. Melanins are a class of structurally diverse pigments that are produced via the oxidation and polymerization of aromatic ring compounds. They share a multitude of remarkable properties such as the ability to absorb ultraviolet (UV) light, transduce electricity, and protect against ionizing radiation [11,12,13]. Unlike most melanotic microorganisms that produce 1,8-dihydroxynaphthalene (DHN) melanin from endogenously synthesized substrates, *C. neoformans* synthesizes 3,4-dihydroxyphenylalanine (DOPA) melanin (also called eumelanin) exclusively from exogenous precursors, particularly from catecholamines and other phenolic compounds found in its environment [14]. As a result, *C. neoformans* has a survival advantage in any niche that is rich in these substrates, a notable example being the human brain, which has a particularly high content of the diphenolic compounds that lead to robust melanin formation [15,16]. Production of this pigment is correlated with resistance to antifungal therapeutics and a high mortality rate [17,18].

Melanization in *C. neoformans* is thought to proceed by deposition of intracellularly synthesized melanin pigments into the cell wall, where they associate with other constituents that are essential for both melanin pigment deposition and retention [19]. The most well-established components of this melanization “scaffold” are the cell-wall polysaccharide chitin, a β-1,4-linked linear polymer of N-acetylglucosamine (GlcNAc) units and chitosan, its deacetylated derivative [20,21]. The deacetylation of cell-wall chitin into chitosan is particularly critical; in the absence of chitosan, cells display a “leaky melanin” phenotype. Although melanin production is unaffected, the pigments do not anchor to the cell wall and consequently leak out into the culture media [22,23]. These findings implicate chitosan as a scaffold for pigment deposition and retention.

A structural role for chitosan in *C. neoformans* melanization is also suggested by solid-state NMR spectroscopy (ssNMR) studies of isolated melanin pigment samples [21,24]. The region of the cell wall containing melanin is highly refractory to degradation and so can be partially purified through a multi-step process in which other cell components are degraded with harsh chemical treatments, but the pigment remains [25]. These preparations of the melanized cell wall are known as melanin “ghosts” due to their yeast-like shape when viewed under a light microscope [26]. A variety of ssNMR techniques have been employed to analyze these samples, demonstrating that the melanin pigments produced by *C. neoformans* are indeed strongly associated with both chitin and chitosan [21]. However, the pigments are not exclusively attached to polysaccharides; several other cellular constituents have also been identified in this melanization scaffold, including substantial quantities of neutral lipids, which are primarily triglycerides [27].

*Saccharomyces cerevisiae* is an ascomycete yeast that is evolutionarily distant from *C. neoformans*. *S. cerevisiae* lacks melanin in its cell wall. However, nitrogen starvation in conjunction with the absence of a suitable carbon source induces the formation of ascospores [28,29], which elaborate a cell wall (the “spore wall”) that contains an aromatic polymer composed of the di-amino acid N,N-bis-formyldityrosine (hereafter, dityrosine) [30,31]. Spore walls are composed of four distinct layers; dityrosine forms the outermost layer of the spore wall and has been suggested to function similarly to melanin in other fungal walls. Underneath the dityrosine layer is a layer consisting primarily of chitosan [32], which is essential for the incorporation of dityrosine into the spore wall [33], suggesting that the chitosan serves as a scaffold for assembly of the dityrosine polymer, analogous to the role of chitosan as an essential scaffold constituent for melanin in the *C. neoformans* cell wall.

Similar to melanin ghosts, the chitosan- and dityrosine-containing outer spore wall has been partially purified and analyzed by ssNMR [34]. These studies have confirmed the presence of both chitin/chitosan and dityrosine but also identified a third component, termed “Chi”. However, the chemical nature of Chi was not determined.

In this report, we use both one-dimensional (1D) and two-dimensional (2D) ssNMR to compare isotopically ^13^C-enriched samples of *S. cerevisiae* spore walls and *C. neoformans* melanin ghosts. Our studies reveal that the cell-wall isolates from the two species are remarkably similar. We demonstrate that the previously unidentified component Chi of the *S. cerevisiae* spore wall is likely to be triglycerides, as are present in *C. neoformans* melanin ghosts. Conversely, the *C. neoformans* melanized wall was found to contain a tyrosine-derived constituent that is not dityrosine and is distinct from the melanin pigment. The similar composition of the cell walls from such phylogenetically distant species leads to the hypothesis that the common chitosan, triglyceride, and polyphenol components of these walls are basic building blocks for a type of fungal cell wall that could exist broadly among other fungal species.

## 2. Materials and Methods

### 2.1. S. cerevisiae Spore Growth Conditions

*S. cerevisiae* AN120 strain cells maintained on yeast extract peptone dextrose agar plates were obtained from a single colony, transferred onto another YPD plate, and grown overnight at 30 °C. The cells were inoculated into 50 mL of synthetic complete media containing a yeast nitrogen base w/o ammonium sulfate and w/o amino acids (US Biologicals, Salem, MA, USA) (1.7 g/L), to which the following reagents were added: dropout amino-acid mix w/o tyrosine or glycine (2 g/L) (US Biologicals, Salem, MA, USA), ^15^N_2_ ammonium sulfate (99%, 5 g/L) (Cambridge Isotope Laboratories, Andover, MA, USA), and [U-^13^C_6_]-D-glucose (99%, 20 g/L) (Cambridge Isotope Laboratories, Andover, MA, USA). Upon reaching the stationary phase, the cells were harvested, washed once with sterile water, and adjusted to a concentration of ~1.85 × 10^7^ mL^−1^ in 2% potassium acetate sporulation medium. The cells were incubated for 2 days at 30 °C prior to being harvested for isolation of the outer spore walls.

### 2.2. Spore Cell Wall Sample Preparation

*S. cerevisiae* AN120 sporulated cells grown using the culture conditions outlined in the previous section were subjected to the outer spore wall isolation protocol as described in detail by Lin et al. (2013). Briefly, the ascospores from ~25 mg (wet weight) of sporulated cells were obtained by enzymatic and chemical disruption of asci followed by centrifugation to remove ascal debris. The spores were separated from vegetative cells using a Percoll (MP Biomedicals, Santa Ana, CA, USA) step gradient and lysed via bead beating, resulting in spore wall fragments, which were separated from any residual intact spores using another Percoll step gradient. These fragments were then further subjected to a series of chemical and enzymatic steps to hydrolyze the proteins and polysaccharides comprising the inner spore wall layers. The remaining outer cell walls were collected via centrifugation, washed several times with water, and dried in a speed vacuum concentrator under low temperature.

### 2.3. C. neoformans Cell Growth Conditions

*C. neoformans* H99 strain (serotype A) cells maintained in frozen glycerol stock were pre-cultured in Sabouraud dextrose broth and incubated at 30 °C with moderate shaking (120 rpm). After 48 h, the cells were harvested, washed once with phosphate buffered saline, counted using a hemocytometer, and adjusted to a concentration of 1 × 10^6^ mL^−1^ in a chemically defined minimal medium (MM) (29.4 mM KH_2_PO_4_, 10 mM MgSO_4_, 13 mM glycine, 3 μM thiamine, 15 mM glucose, pH 5.5) containing l-DOPA at a 1 mM concentration as the obligatory exogenous melanization precursor. To produce cells in which only the non-melanin cellular constituents are uniformly enriched in the ^13^C isotope, the cell culture was prepared in MM containing [U-^13^C_6_]-glucose as the sole carbon source. To selectively ^13^C-enrich only the tyrosine-derived moieties, the cell culture was prepared in MM containing glucose at natural abundance and supplemented with [U-^13^C_9_]-tyrosine at a 0.5 mM concentration. Each cell culture was incubated for 10 days at 30 °C with shaking at moderate speed (120 rpm) prior to being harvested for isolation of the melanized cell walls (melanin “ghosts”).

### 2.4. Melanin Ghost Sample Preparation

*C. neoformans* H99 cells grown using the culture conditions outlined in the previous section were subjected to the melanin ghost isolation protocol, which has been described in our prior studies [35]. Briefly, cells obtained from 100 mL of melanized culture were subjected to enzymatic lysis of cell-wall polysaccharides, chemical denaturation and subsequent enzymatic hydrolysis of protein constituents, three consecutive Folch lipid extractions using a 3:4:8 solvent ratio of saline:methanol:chloroform, and 1 h boiling in 6 M HCl. The solid black particles that “survive” this process were obtained via centrifugation, dialyzed against water, and lyophilized.

### 2.5. Solid-State NMR Spectroscopy

Experiments for outer spore cell wall samples isolated from *S. cerevisiae* were carried out on either 600 or 750 MHz Bruker AVANCE spectrometers equipped with 4 mm HX magic angle spinning (MAS) probes. The data were acquired on ~50–100 mg of dry material using a MAS rate of 10.00 ± 0.01 kHz and a spectrometer-set temperature of 25 °C. The 1D ^13^C cross-polarization (CPMAS) and 2D ^13^C-^13^C double-quantum (DQ) ^13^C-^13^C correlation experiments were conducted on the 600 MHz spectrometer using ^13^C and ^1^H 90° pulse lengths of 4.0 and 3.0 μs, respectively. The CPMAS experiment was conducted using a 20% linearly ramped amplitude for ^1^H and a 2 ms contact time for ^1^H-^13^C polarization transfer. The DQ ^13^C-^13^C correlation experiment was conducted using the refocused J-INADEQUATE pulse sequence [36,37] and 3.0 ms spin echo delays. For both experiments, two-pulse phase-modulated (TPPM) decoupling [38] with a radio frequency field of 88 kHz was applied during signal acquisition; the ^13^C chemical shifts were referenced externally to the carbonyl carbon signal of glycine, which resonates at 176.5 ppm on the tetramethylsilane scale. The 2D ^13^C-^13^C through-space dipolar assisted rotational resonance (DARR) correlation experiment [39,40] was conducted on the 750 MHz spectrometer using ^13^C and ^1^H 90° pulse lengths of 5.0 and 2.9 μs, respectively, and a 500 ms mixing time. TPPM decoupling was applied during signal acquisition with a radio frequency field of 83 kHz. The ^13^C chemical shifts were referenced externally to the methylene carbon signal of adamantane at 38.5 ppm on the tetramethylsilane scale.

Experiments for the melanized cell wall (melanin “ghost”) samples isolated from *C. neoformans* were carried out on a 600 MHz Varian (Agilent) DirectDrive2 spectrometer equipped with a 3.2 mm T3 HXY MAS probe. The data were acquired on ~12–18 mg of lyophilized material using a MAS rate of 15.00 ± 0.02 kHz and a spectrometer-set temperature of 25 °C. All experiments were conducted with ^13^C and ^1^H 90° pulse lengths of 2.6 and 2.3 μs, respectively. The CPMAS experiment was conducted using a 20% linearly ramped amplitude for ^1^H and a 2 ms contact time for ^1^H-^13^C polarization transfer. The DQ ^13^C-^13^C correlation experiment was conducted using the refocused J-INADEQUATE pulse sequence and 2.4 ms spin echo delays. The ^13^C-^13^C DARR experiment carried out for the ^13^C-glucose-enriched melanin ghost sample was acquired using a 500 ms mixing time; the DARR experiment carried out for the melanin ghost sample generated from ^13^C-tyrosine-supplemented *C. neoformans* cells was acquired using a 50 ms mixing time. For all experiments, small phase incremental alternation pulse sequence (SPINAL) decoupling [41] was applied during signal acquisition with a radio frequency field of 83 kHz. The ^13^C chemical shifts were referenced externally to the methylene carbon signal of adamantane at 38.5 ppm.

## 3. Results

### 3.1. The ^13^C CPMAS ssNMR Spectra of S. cerevisiae Spore Walls and C. neoformans Melanin “Ghosts” Display Several Similarities that Suggest Common Structural Elements

**Figure 1:**
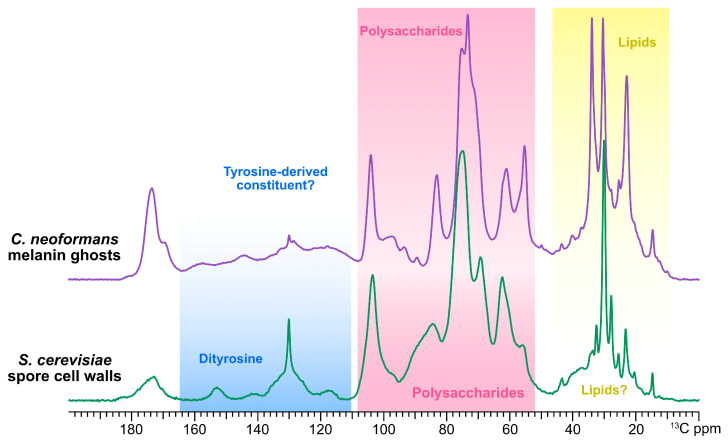
One-dimensional (1D) ^13^C CPMAS solid-state NMR (ssNMR) spectral comparison of outer spore walls isolated from wild type (AN120) *S. cerevisiae* spore cell walls and melanin ghosts isolated from wild type (H99) *C. neoformans* cells. The cells from both species were grown in media containing [U-^13^C_6_]-enriched glucose as the sole carbon source. *C. neoformans* cells were also provided natural abundance l-DOPA and thus signals attributable to the melanin pigment are not observed. The spectral region shaded in blue displays the peaks previously attributed to dityrosine carbons in *S. cerevisiae* spore cell walls, and the region in yellow to lipid carbons in *C. neoformans* melanin ghosts. The spectral region shaded pink displays peaks previously attributed to the carbons of the polysaccharides in each sample. The unshaded spectral region (165–185 ppm) displays several overlapping peaks that are attributed to the carbonyl carbons in all three constituent types.

This work was inspired by a serendipitous observation of similarities between the cross-polarization magic-angle spinning (CPMAS) ^13^C ssNMR spectra of outer spore walls isolated from *S. cerevisiae* [34] and melanized cell walls (melanin “ghosts”) isolated from *C. neoformans* [21]. Figure 1 compares previously reported 1D CPMAS ^13^C ssNMR spectra for the *S. cerevisiae* outer spore wall with comparably acquired data for the melanin scaffold of the *C. neoformans* wall, demonstrating the close correspondence of their chemical shifts and the relative intensities of their respective resonances.

As described previously by Lin et al. (2013), the CPMAS ^13^C spectrum of the *S. cerevisiae* outer spore cell wall displays peaks attributable to at least three different constituents: (1) the cell-wall polysaccharide carbons, primarily chitin and chitosan (~55–105 ppm, shaded pink); (2) broad resonances in the 110–160 ppm spectral region (shaded blue) attributed to the aromatic carbons of dityrosine phenol rings, identified from both their ^13^C chemical shifts and the analysis of spore walls from mutant strains; and (3) several prominent peaks between 10 and 40 ppm (shaded yellow) that did not correspond to carbons of any known spore cell wall moiety and were thus attributed to an unidentified component designated as Chi.

The 1% natural abundance ^13^C NMR spectrum of melanized cell walls is typically complicated by an overlap of the melanin pigment signals and the signals of other wall components [35,42]. However, since *C. neoformans* is unable to synthesize melanin pigments from nutrient sources such as glucose [43], cultures grown with a natural abundance ^13^C-containing melanization precursor (e.g., l-DOPA) and stable isotope-labeled [U-^13^C_6_]-glucose as the sole carbon source yield cells in which only non-pigment moieties are enriched with the NMR-active ^13^C isotope. As a result, the ^13^C ssNMR spectra of melanin ghosts prepared from ^13^C-enriched glucose cultures, illustrated in Figure 1, display signals attributable solely to those glucose-derived constituents that survive the degradative isolation protocol. The absence of signals from the DOPA-derived pigment allows the unambiguous identification of the cellular constituents that make up this melanin scaffold. In particular, the signals shaded in pink have been shown to arise from chitin and chitosan polysaccharides [21], whereas those shaded in yellow have been attributed to the methyl and methylene carbons within the fatty acyl chains of triglycerides [27].

The spectral similarity of the two different yeast cell walls is striking. Whereas the correspondence of the respective polysaccharide regions is to be expected given that both walls are known to contain chitin and chitosan, the nearly identical chemical shifts and similar intensities in the region from 10 to 40 ppm suggest the presence of lipids in the *S. cerevisiae* outer spore cell wall that are similar in molecular structure to the *C. neoformans* melanized wall. Conversely, the spectral region between 110 and 160 ppm in the *C. neoformans* sample includes several broad resonances that have not previously been assigned. These latter peaks bear a noticeable resemblance to features in the analogous region of the *S. cerevisiae* CPMAS ^13^C NMR spectrum, which have been attributed to dityrosine. Considering that these aromatic carbon peaks cannot originate from the melanin pigment and must instead arise from a glucose-derived constituent, we considered that the *C. neoformans* cell wall could contain a previously unidentified “dityrosine-like” molecular moiety.

### 3.2. S. cerevisiae Spore Cell Walls and C. neoformans Melanin Ghosts Are both Comprised of Chitinous Polysaccharides, Tyrosine-Derived Constituents, and Neutral Lipids

**Figure 2:**
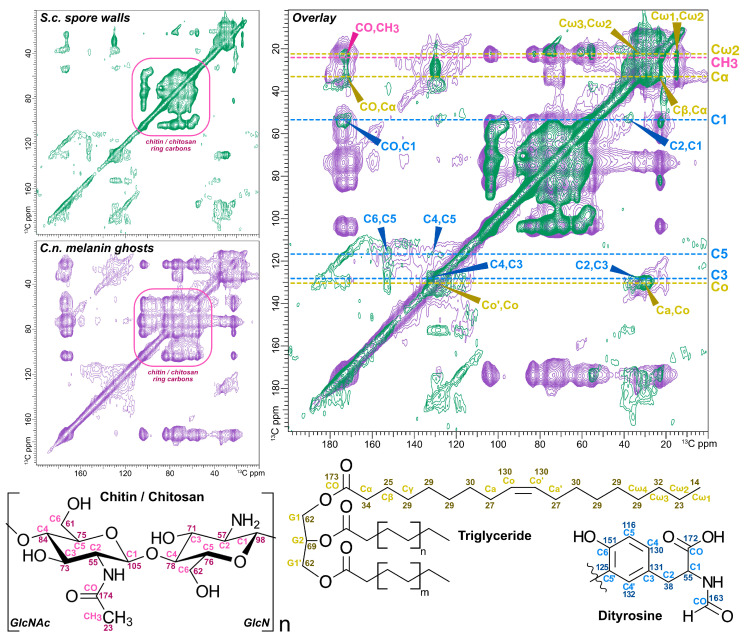
Two-dimensional (2D) ^13^C-^13^C dipolar assisted rotational resonance (DARR) ssNMR spectral comparison of outer spore walls isolated from wild type (AN120) *S. cerevisiae* cells (green spectrum) and melanin ghosts isolated from wild type (H99) *C. neoformans* cells (purple spectrum), each grown in media containing ^13^C-enriched glucose as the sole carbon source. The experiments were conducted using a 500 ms mixing time and therefore display “through-space” correlations between carbon pairs up to ~6 Å apart from one another. Specific carbon resonances are indicated in the overlay of the two spectra. Pink indicates carbons from chitin; blue from dityrosine; yellow from triacylglycerol. Numbers on the structures at the bottom indicate the expected chemical shift for each carbon.

The shared 1D CPMAS ^13^C NMR spectral features of outer spore walls isolated from *S. cerevisiae* and melanin ghosts from *C. neoformans* prompted us to devise more stringent tests for molecular-level structural similarities between these two disparate types of yeast preparations. To begin our investigation, [U-^13^C_6_]-glucose-enriched samples of *S. cerevisiae* spore cell walls and *C. neoformans* melanin ghosts were examined using 2D ^13^C-^13^C dipolar assisted rotation recoupling (DARR) solid-state NMR experiments. A relatively long mixing time of 500 ms was used to detect proximal pairs of ^13^C-^13^C nuclei located up to ~6 Å (as much as three bond lengths) distant in space from one another. This strategy serves to spread the spectral features into a second spectroscopic dimension and moreover, to identify nearby pairs of carbons within the macromolecular assemblies. Whereas *C. neoformans* melanin ghosts have been studied extensively using a variety of homo- and heteronuclear multi-dimensional ssNMR techniques [24,27,42,44,45], only 1D ^13^C ssNMR data have been reported for *S. cerevisiae* spore walls [34]. Thus, the comprehensive identifications made previously for melanin ghosts were used to guide spectroscopic analysis of the 2D DARR results for spore walls. Overall, a side-by-side examination of the *S. cerevisiae* spore wall and *C. neoformans* melanin ghost DARR contour plots (Figure 2) revealed that the majority of the signal intensity displayed in each spectrum is attributable to the same three constituent types—polysaccharides, polyaromatics, and lipids—albeit with some variations within each structural type.

As observed for melanin ghosts, correlations corresponding to pairwise interactions between polysaccharide ring carbons (~55–105 ppm) contribute strongly to the spore wall DARR data, including cross peaks that demonstrate the presence of both chitin and its deacetylated product chitosan [46,47]. For instance, the acetyl group of chitin can be identified unambiguously by the unique ^13^C chemical shifts of the methyl and carbonyl carbons at ~23 and 174 ppm, respectively. The carbons of this covalently bonded pair are correlated with one another (23 × 174 ppm) and also to the nearby sugar ring carbons. Both chitin and chitosan can be distinguished by the 56 ppm chemical shifts of their C2 carbons, which contrast with ~70–80 ppm spectral features observed for most polysaccharides. Nonetheless, chitin and chitosan will each display DARR correlations with different subsets of proximal ring carbons. As the highly deacetylated form of chitosan found in *C. neoformans* melanin ghosts was shown using 2D ^13^C-^15^N ssNMR spectroscopy to include a C1 ring carbon that resonates ~90–100 ppm and a C3 carbon at ~70 ppm, the correlations of C2-C1 and C2-C3 carbon-pairs can be identified at 56 × 97 and 56 × 71 ppm, respectively. In contrast, C2-C1 and C2-C3 correlations for chitin appear at 56 × 104 and 56 × 74 ppm, respectively. The overall concordance of these features in the two DARR spectra indicates that, as in *C. neoformans* melanin ghosts, *S. cerevisiae* spore cell walls contain a highly deacetylated form of chitosan. However, the notably fewer intramolecular correlations observed involving chitin and chitosan carbons for the spore walls suggest differences in the predominance and/or sequential patterns of deacetylated products between the two yeast species [48,49,50].

The two DARR plots also display several common cross-peaks in the 110–160 ppm spectral region that correspond to aromatic and doubly bonded carbons. Although the cross-peaks are exceptionally broad in both spectra, the spectral features observed for the *S. cerevisiae* spore wall can be attributed unambiguously to correlations between proximal carbon pairs within dityrosine, a well-established spore-wall specific constituent [31,34]. In contrast, the pattern of pairwise correlations in the aromatic region of the melanin ghost DARR plot differs from that displayed for the spore walls. This observation argues against assigning the non-melanin aromatic constituent in the *C. neoformans* cell wall to dityrosine. Consistent with this conclusion, *C. neoformans* cells fail to exhibit the blue fluorescence characteristic of dityrosine (A.M.N., unpublished observations).

The final striking similarity witnessed between these 2D spectra pertains to a group of cross-peaks that, in *C. neoformans* melanin ghosts, have been determined to arise from proximal carbon correlations within triglycerides that have unsaturated fatty acid (FA) acyl chains [27]. Although the diagnostic triglyceride glycerol carbon peaks (~62 and 69 ppm) are obscured in this experiment by overlap with polysaccharide ring carbon signals, several key cross-peaks that are hallmarks of unsaturated FAs are clearly identifiable in both DARR spectra [51,52]. For example, a correlation between the carbonyl carbon (CO) and a proximal Cα methylene carbon is observed at 172 × 35 ppm; a correlation between the Cα and Cβ carbons appears at 35 × 25 ppm. Toward the other end of the acyl chain, we can identify cross-peaks from FA carbon pairs at 32 × 23 ppm (Cω_3_-Cω_2_) and 23 × 14 ppm (Cω_2_-Cω_1_) by reference to analogous correlations previously determined for *C. neoformans* melanin ghosts. Moreover, the cross-peak at 27 × 130 ppm represents a correlation between the allylic and olefinic carbons (denoted as Ca and Co, respectively) of an unsaturated FA chain [53,54]. Nonetheless, only the melanin ghost DARR displays additional signals in this spectral region that support more than one point of unsaturation. For example, the cross-peak at 26 × 130 ppm is attributable to the methylene bis-allylic carbon and the two adjoining chemically equivalent olefinic carbons in a di-unsaturated FA such as linoleic acid (C18:2) [53]. Therefore, our data suggest that in contrast to *C. neoformans* melanin ghosts, which contain a mixture of mono- and polyunsaturated FAs, *S. cerevisiae* spore cell walls contain only monounsaturated species such as oleic acid (C18:1).

### 3.3. 2D ^13^C-^13^C ssNMR Implicates Triglycerides as a Component of S. cerevisiae Spore Cell Walls

To complement and strengthen the peak assignments deduced from the through-space DARR data, samples of *S. cerevisiae* spore walls and *C. neoformans* melanin ghosts were examined using the through-bond 2D ^13^C-^13^C J-INADEQUATE experiment. In contrast to DARR, which observes carbon pairs within ~6 Å of one another, the J-INADEQUATE experiment exclusively detects carbon pairs that are directly bonded to one another. The resulting spectra therefore display comparatively fewer cross-peaks and are less likely to suffer from spectral crowding. Moreover, in 2D J-INADEQUATE spectra, each carbon within a covalently bonded pair is represented by a cross-peak with a unique single quantum (SQ) ^13^C chemical shift (x-axis), but both carbons share a common double quantum (DQ) ^13^C chemical shift that is equal to the sum of the two SQ shifts (y-axis). Consequently, spectral overlap is reduced by spreading the SQ resonances over a larger frequency range in an orthogonal direction. Although this experiment has lower sensitivity overall than 2D DARR and disfavors broad spectral features such as the dityrosine and other aromatic moieties, the superior resolution of J-INADEQUATE allowed us to both confirm and extend our determination of molecular structures in these two cell wall types.

**Figure 3:**
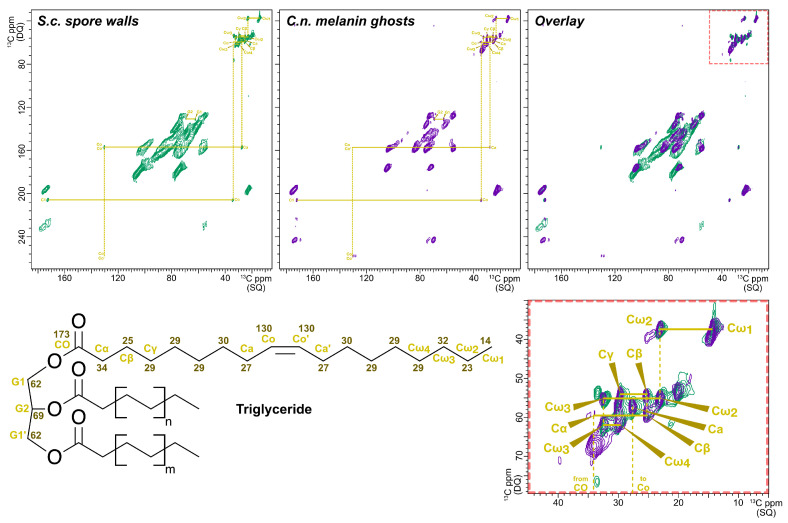
2D ^13^C-^13^C J-INADEQUATE ssNMR spectral comparison of outer spore walls isolated from wild type (AN120) *S. cerevisiae* cells (green spectrum) and melanin ghosts isolated from wild type (H99) *C. neoformans* cells (purple spectrum), both grown in media containing ^13^C-enriched glucose as the sole carbon source. The displayed signals are attributable to pairs of carbons separated by one covalent bond. The cross-peaks corresponding to the connectivities within triglycerides have been annotated with wedges. The box at the lower right shows an expanded region of the overlay (red dashed lines). The numbers on the structure at the bottom left indicate the expected chemical shift for each carbon.

Analogous to the ^13^C ssNMR spectra compared in Figure 1 and Figure 2, the J-INADEQUATE spectra in Figure 3 both display prominent contributions from signals attributable to chitin and chitosan; cross-peaks corresponding to covalent linkages between the ring carbons of each form of this chitinous polysaccharide are readily observed for the cell walls from both species (~55–105 ppm SQ). As in the 2D DARR spectra (Figure 2), the two samples exhibit different degrees of distinction between chitin and chitosan linewidths, which can reflect variations in deacetylation, which influence the rigidity of the chitosan scaffold [21].

The J-INADEQUATE data also indicated that both *S. cerevisiae* spore walls and *C. neoformans* ghosts contain at least one other polysaccharide type in addition to chitin and chitosan. The anomeric (C1) carbons of all polysaccharides resonate at approximately 100 ppm, a frequency value ~20 ppm greater than observed for the other ring carbons. Chitin and chitosan have characteristic C2 carbon chemical shifts of ~56–57 ppm; thus, the two pairs of SQ signals (104 and 56 ppm; 97 and 57 ppm) correlated with DQ frequencies of 160 ppm and 154 ppm are attributed to the C1-C2 linkages within chitin and chitosan, respectively. However, both J-INADEQUATE spectra display a pair of cross-peaks at 105 and 72 ppm that correspond to a C1-C2 linkage within a non-nitrogenous polysaccharide such as a *β*-glucan.

The J-INADEQUATE spectra also offer important new evidence for triglycerides that are present in both *S. cerevisiae* spore walls and *C. neoformans* melanin ghosts. Notably, several through-space correlations that were obscured by overlapping signals in the DARR spectra are now revealed and can be identified with triglyceride carbons by reference to prior data for melanin ghosts [21]. In particular, the pair of SQ signals at 62 and 69 ppm are correlated to produce a DQ sum at 131 ppm. These signals represent the covalently linked CHO with two CH_2_O groups of a glycerol backbone [55,56,57,58]; because each of the three carbons is esterified to a similar type of FA, the resulting symmetrical molecule gives rise to only two inequivalent resonances. As anticipated, the J-INADEQUATE experiment also confirms covalently bonded carbon pairs of the long acyl chains e.g., CO-Cα (172 and 35 ppm) and Cω_2_-Cω_1_ (23 and 14 ppm). Ultimately, every unique triglyceride ^13^C resonance observed in the J-INADEQUATE experiment for spore walls has previously been identified in melanin ghosts using ssNMR spectral editing techniques that select for signals from mobile constituents [21]. Thus, taken together, 2D DARR and J-INADEQUATE offer strong evidence in *S. cerevisiae* spore walls for each unique triglyceride carbon resonance identified previously by ssNMR in melanin ghosts and indicate that the material previously designated Chi is actually triglycerides.

### 3.4. Exogenously Provided ^13^C-Enriched Tyrosine is Incorporated into the C. neoformans Melanized Cell Wall as Non-Pigment Constituent

In light of our observation that the 2D DARR spectrum of *C. neoformans* melanin ghosts displays ^13^C-^13^C correlations similar to those attributed to dityrosine in *S. cerevisiae* spore walls (Figure 2), we postulated that a previously unidentified “dityrosine-like” constituent could be present in the *C. neoformans* cell wall and be retained in melanin ghosts. To test this proposal, *C. neoformans* cells grown in media containing a natural abundance carbon source and a melanization precursor were supplemented with [U-^13^C_9_]-enriched tyrosine and the resulting melanin ghost samples were examined using ^13^C ssNMR spectroscopy. We hypothesized that the observation of signals consistent with tyrosine carbons would indicate that a tyrosine-derived moiety was present in the melanized cell wall for the following two reasons: (1) *C. neoformans* melanin synthesis is catalyzed by a laccase enzyme [59], which precludes the utilization of tyrosine as a melanization precursor [43]. Laccase, unlike other melanization-catalyzing polyphenol oxidases such as tyrosinase, has little affinity for monophenolic compounds and cannot convert them into the diphenolic substrates required to carry out the initial oxidation step of melanin synthesis [60,61,62]. (2) Similarly to *S. cerevisiae*, *C. neoformans* lacks the enzymatic machinery required for aromatic ring cleavage, which also precludes the utilization of tyrosine as a primary source of nutritional fuel [43,63]. Together, these metabolic characteristics suggest that the exogenous ^13^C-tyrosine would be incorporated largely intact into tyrosine-containing moieties and the ^13^C carbons would not be metabolized into other molecules.

**Figure 4:**
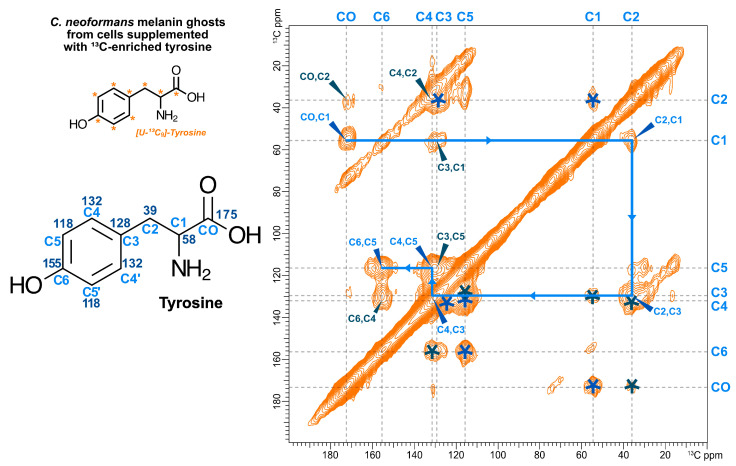
2D ^13^C-^13^C DARR spectrum (50 ms mixing time) of *C. neoformans* melanin ghosts generated from a cell culture supplemented with 0.5 mM [U-^13^C_9_]-tyrosine that contained natural abundance glucose and l-DOPA as the sole carbon source and obligatory melanization precursor, respectively. The cross-peaks displayed correspond to proximal carbon pairs within tyrosine separated by 1 bond (blue) or 2 bonds (navy). Each ^13^C-^13^C pair is annotated with its carbon assignment on one side of the diagonally symmetrical DARR plot and with an asterisk on the other. The blue arrowed line highlights the one-bond connectivities within tyrosine that indicate the molecule did not undergo any chemical modifications. The numbers on the structure at the left indicate the expected chemical shift for each carbon.

The 2D ^13^C-^13^C DARR spectrum of melanin ghosts generated from [U-^13^C_9_]-tyrosine-supplemented *C. neoformans* cells is shown in Figure 4. Several insights can be derived from these data. First, the presence of DARR cross-peaks suggests that the exogenously provided [U-^13^C_9_]-tyrosine was indeed taken up by *C. neoformans* cells. Given that the experiment we conducted was designed to detect proximal pairs of ^13^C-^13^C nuclei separated by distances corresponding to ~1–2 bond lengths, such pairs can only be observed if both partners are isotopically enriched rather than present at natural abundance (~1.1% each). Secondly, the [U-^13^C_9_]-tyrosine-supplemented ghost DARR spectrum displays only the few cross-peaks that can be attributed to intramolecular correlations between tyrosine carbons, validating our supposition that the ^13^C-enriched tyrosine is spared from degradation as an energy source and will be incorporated “as is”. Third, the cross-peaks between the carbons at ~38 and 57 ppm correspond to correlations between the side chain C1 and C2 carbons of uncyclized tyrosine moieties, indicating that the ^13^C-labeled compounds taken up by *C. neoformans* cells are not transformed into indoles. Since the melanin pigments produced by *C. neoformans* are primarily composed of indolic compounds (i.e., cyclized phenolic compounds), the observation of exclusively open-chain carbon cross-peaks supports the claim that the exogenously provided labeled compound is not used for melanin synthesis and corroborates the inability of this organism to use tyrosine as a precursor for melanin formation. Lastly, the observed chemical shifts match those found in DARR spectra of the ^13^C-glucose ghosts and deviate slightly from those of the dityrosine peaks in the *S. cerevisiae* spore wall DARR (Figure 2), indicating that the labeled material is present in the form of tyrosine rather than dityrosine. Together, these observations indicate that tyrosine is a constituent of the *C. neoformans* cell wall and thus part of the melanin scaffold.

## 4. Discussion

Our spectroscopic results show a remarkable molecular similarity between the melanized portion of the *C. neoformans* cell wall and the outer layers of the *S. cerevisiae* spore wall. Both assemblies rely on the deacetylation of chitin to form chitosan, which functions as a scaffold for the deposition of a polyphenolic component, melanin or dityrosine, as well as for neutral lipids in the form of triglycerides.

The identification of the Chi constituent of the spore wall as a neutral lipid is particularly noteworthy, as it is consistent with several earlier cytological and genetic observations [64]. Within the cell cytoplasm, triglycerides are stored in lipid droplets [65]. Ascospores are formed within the cytoplasm of the parent cell (called an ascus) in a manner that exposes the developing spore wall to the ascal cytoplasm [66]. Recent studies have revealed that a class of lipid droplets is associated with the developing spore wall [34,67,68]. Thus, these lipid droplets might provide a source for the triglycerides incorporated in the spore wall. Moreover, mutants that block the synthesis of triglycerides show spore wall defects, in particular reduced staining with the chitosan-specific dye eosin Y, suggesting that triglycerides might be important for proper construction of the chitosan layer of the spore wall [69].

The lipid droplets associated with the outer spore wall carry several proteins including the cis-prenyltransferase Srt1 [68]. Loss of this enzyme, which synthesizes a lipid-droplet localized pool of polyisoprenoids, results in loss of the chitosan and dityrosine layers of the spore wall [70]. This property provides an additional link between the lipid droplets and spore wall assembly, and it might also offer an additional parallel between spore walls and melanin ghosts; although polyisoprenoids are not evident in our ssNMR spectra of the *S. cerevisiae* outer spore wall, they have recently been identified in *C. neoformans* melanin ghosts through the implementation of ssNMR experiments that select for mobile constituents such as lipids [27,71].

In our earlier work on Srt1, we proposed that the polyisoprenoids synthesized by Srt1 might act as signals to induce production of the chitin necessary to form the spore wall chitosan layer [70]. The results reported here suggest an alternative possibility; that triglycerides, and perhaps other lipids, are components of the wall structure itself. This raises the important question of how such highly hydrophobic molecules are incorporated into the wall. Notably, the melanin ghost isolation protocol includes a step where three successive lipid extractions are performed using a saline:methanol:chloroform solvent mixture for lipid removal, suggesting that the residual triglycerides are somehow anchored within the cell wall. Conversely, ssNMR analysis has demonstrated that the triglycerides within these cell-wall preparations undergo molecular motions consistent with freely mobile molecules, and therefore are unlikely to be covalently linked to other wall components [27]. The standard protocol used to isolate *S. cerevisiae* outer spore walls does not include a lipid extraction step. However, subjecting sporulated *S. cerevisiae* cells to the melanin ghost protocol yields cell wall samples that are suggested by preliminary ssNMR experiments to be comparable to those obtained with traditional spore wall isolation and indeed contain substantial quantities of mobile lipids (C.C. unpublished obs.). It is not yet clear how to resolve this paradox. One industrial use of chitosan is in the synthesis of nanoparticles, for example for drug delivery, in which a core of hydrophobic molecules is protected by a chitosan coating [72,73,74]. Though speculative, a similar structural organization—a core of neutral lipids encapsulated by chitosan strands—could account for the concurrent mobility and inextractability of the triglycerides in the spore wall preparations.

Our study also reveals that the melanized cell wall contains a tyrosine-derived non-melanin component. Although not dityrosine itself, this finding may represent another parallel between the melanin ghosts and the outer spore wall; genetic studies in *S. cerevisiae* have shown that blocking de novo tyrosine synthesis has a stronger effect on spore wall function than blocking dityrosine synthesis [75], suggesting that tyrosine promotes spore wall function independently of its role as a precursor to dityrosine. The possible function of tyrosine in the *C. neoformans* and *S. cerevisiae* walls remains to be determined.

In sum, the results presented herein demonstrate that the highly degradation-resistant materials from the cell walls of melanized *C. neoformans* and *S. cerevisiae* spores share a common set of components: a polyphenol polymer (melanin or dityrosine); chitosan; and triglyceride lipids. Given that the ascomycetes and basidiomycetes separated from 400 million to 1.8 billion years ago depending on the clock used to calculate evolutionary divergence, our results suggest conservation of basic cell wall structures despite the spatial and temporal separation in deep time [76]. The presence of these common components suggests that they represent a “tool kit” used to construct stress resistant cell walls, which are broadly conserved in fungi.
